# Probiotic-Derived Polyphosphate Enhances the Epithelial Barrier Function and Maintains Intestinal Homeostasis through Integrin–p38 MAPK Pathway

**DOI:** 10.1371/journal.pone.0023278

**Published:** 2011-08-15

**Authors:** Shuichi Segawa, Mikihiro Fujiya, Hiroaki Konishi, Nobuhiro Ueno, Naoyuki Kobayashi, Tatsuro Shigyo, Yutaka Kohgo

**Affiliations:** 1 Frontier Laboratories of Value Creation, Sapporo Breweries Ltd., Yaizu, Japan; 2 Division of Gastroenterology and Hematology/Oncology, Department of Medicine, Asahikawa Medical University, Asahikawa, Japan; Charité-University Medicine Berlin, Germany

## Abstract

Probiotics exhibit beneficial effects on human health, particularly in the maintenance of intestinal homeostasis in a complex manner notwithstanding the diversity of an intestinal flora between individuals. Thus, it is highly probable that some common molecules secreted by probiotic and/or commensal bacteria contribute to the maintenance of intestinal homeostasis and protect the intestinal epithelium from injurious stimuli. To address this question, we aimed to isolate the cytoprotective compound from a lactobacillus strain, *Lactobacillus brevis* SBC8803 which possess the ability to induce cytoprotective heat shock proteins in mouse small intestine. *L. brevis* was incubated in MRS broth and the supernatant was passed through with a 0.2-µm filter. Caco2/bbe cells were treated with the culture supernatant, and HSP27 expression was evaluated by Western blotting. HSP27-inducible components were separated by ammonium sulfate precipitation, DEAE anion exchange chromatography, gel filtration, and HPLC. Finally, we identified that the HSP27-inducible fraction was polyphosphate (poly P), a simple repeated structure of phosphates, which is a common product of lactobacilli and other bacteria associated with intestinal microflora without any definitive physiological functions. Then, poly P was synthesized by poly P-synthesizing enzyme polyphosphate kinase. The synthesized poly P significantly induced HSP27 from Caco2/BBE cells. In addition, Poly P suppressed the oxidant-induced intestinal permeability in the mouse small intestine and pharmacological inhibitors of p38 MAPK and integrins counteract its protective effect. Daily intrarectal administration of poly P (10 µg) improved the inflammation grade and survival rate in 4% sodium dextran sulfate-administered mice. This study, for the first time, demonstrated that poly P is the molecule responsible for maintaining intestinal barrier actions which are mediated through the intestinal integrin β1-p38 MAPK.

## Introduction

The human gastrointestinal tract is colonized by a vast community of bacteria. Well-balanced interactions between the host and gut microbiota provides health benefits for the body [1]. Probiotics in particular deliver beneficial effects including competitive exclusion of pathogenic bacteria [2], induction of defensin production [3], [4], modulation of host immune functions [5], and improvement of the intestinal barrier function [6]. Although these actions appear to be mediated by soluble factors secreted from probiotics [7]–[12], the molecules responsible for the probiotic effects have rarely been identified [7], [8]. We previously found that competence and sporulation factor, a pentapeptide produced by *Bacillus subtilis*, exerts a cytoprotective effect by inducing heat shock proteins and activating the p38 mitogen-activated protein kinase (MAPK) and phosphatidylinositol 3-kinase (Akt) pathways [7]. Subsequently, soluble proteins p75 and p40 produced by *Lactobacillus rhamnosus* GG (LGG) were identified as functional molecules possessing inhibitory effects on inflammatory cytokine-induced cell apoptosis by activating the Akt pathway [8]. The identification of these three molecules, which are specifically produced by *B. subtilis* and LGG helped us to elucidate the probiotic mechanisms of actions.

Intestinal homeostasis, on the other hand, is maintained in a complex manner notwithstanding the diversity of an intestinal flora between individuals. Thus, it is highly probable that some common molecules secreted by probiotic and/or commensal bacteria contribute to the maintenance of intestinal homeostasis and protect the intestinal epithelium from injurious stimuli. To address this question, we selected a lactobacillus strain, *Lactobacillus brevis* SBC8803 which possess the ability to induce cytoprotective heat shock proteins in mouse small intestine, protect intestinal tissues from oxidant stress and improve the intestinal injury in a mouse colitis model [13]. We tried to isolate the cytoprotective compound from the culture supernatant of *L. brevis* SBC8803. In this study, we proposed for the first time that polyphosphate (poly P), a common product of lactobacilli and other bacteria associated with intestinal microflora, is the molecule responsible for probiotic actions. In addition, we revealed that intestinal integrin β1mediated the bacteria-derived poly P functions including enhancement of intestinal barrier function and improvement of intestinal injury in mouse colitis model.

## Results

### Induction of cytoprotective HSP27 by the culture supernatant of *L. brevis* SB8803 in Caco2/BBE cells

**Figure 1:**
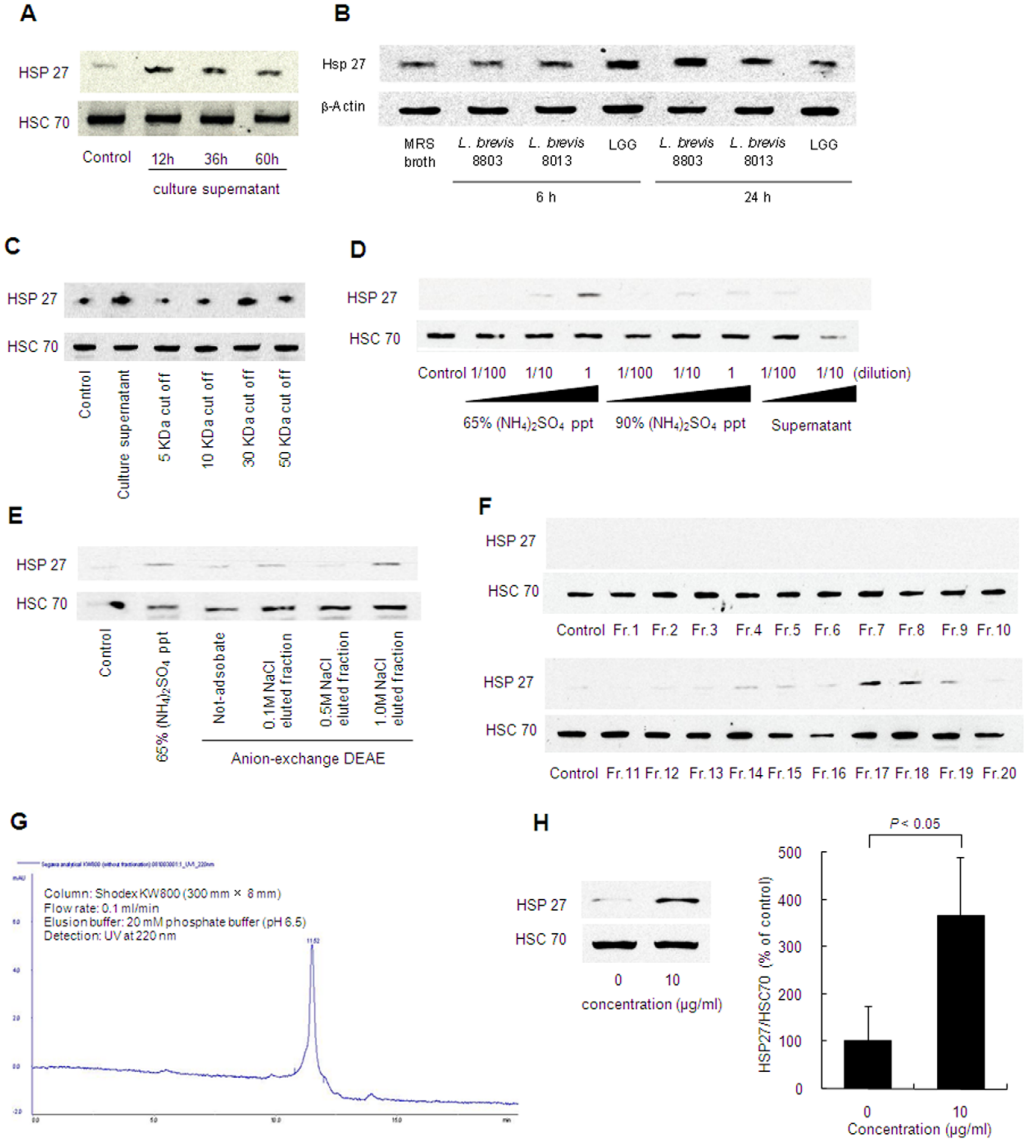
HSP27-inducible substance was separated from *L. brevis* SBC8803 culture supernatant. (A) HSP27 induction from Caco2/BBE cells by culture supernatant obtained from the *L. brevis* 8803 culture broth at 12, 36 and 60 h. (B) HSP27 induction from Caco2/BBE cells by culture supernatant of *L. brevis* 8803, *L. brevis* 8013 and LGG obtained from the bacterial culture broth at 6 and 24 h. (C) HSP27 induction by filtrate of *L. brevis* SBC8803 culture supernatant passed through a 5-, 10-, 30- or 50-kDa membrane. (D) HSP27 induction by 65% or 90% ammonium sulfate precipitate of *L. brevis* SBC8803 culture supernatant. (E) HSP27 induction by fraction separated from 65% ammonium sulfate precipitate by DEAE anion-exchange chromatography. (F) HSP27 induction by fraction separated from 0.1 M NaCl-eluted fraction by size-exclusion chromatography. (G) HPLC chromatogram of the HSP-inducible fraction. Sample was separated on a Shodex KW800 column (300 mm×8 mm ID), eluted with 20 mM phosphate buffer (pH 6.5) at a flow rate of 0.1 mL/min. The eluent was monitored with an ultraviolet spectrophotometer at 220 nm. (H) HSP27 induction by 10 µg/mL of HSP27-inducible fraction (n = 3).

Human intestinal epithelial Caco2/BBE cells were stimulated with the culture supernatant of *L. brevis* SBC8803 *in vitro* to elucidate HSP27 induction. The culture supernatant was eluted by filtration with a 0.2 µm filter to completely remove the bacterial body from culture medium. HSP27 was induced by the culture supernatant while constitutive HSC70 expression remained unchanged. HSP27 induction was affected by the bacterial culture time (Figure 1A). The supernatant obtained at 12 h from bacterial culture (0.11×10^9^ cfu/mL) strongly induced HSP27 compared with that obtained at 36 h (1.07×10^9^ cfu/mL) and 60 h (0.95×10^9^ cfu/mL). Similar results were obtained in the case of LGG and *L. brevis* SBC8013 (Figure 1B).

### Identification of the molecule responsible for the induction of HSP27

**Figure 2:**
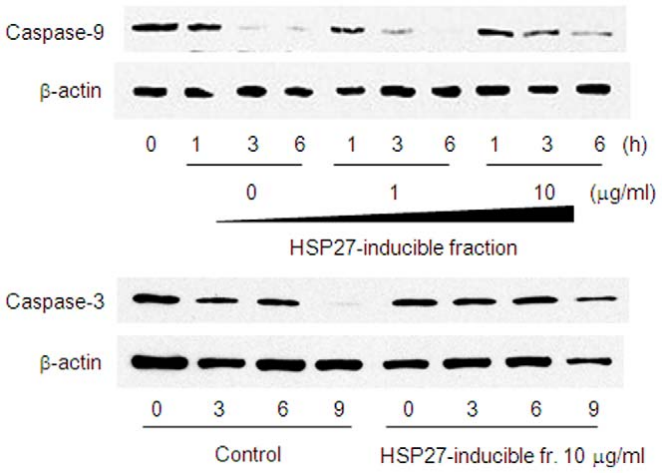
Anti-apoptopic property of HSP27-inducible fraction. Time course of inactive full-length caspase-3 and -9 degradation in Caco2/BBE induced by 1 µM staurosporine treatment in the presence or absence of HSP27-inducible fraction.

To help identify the molecule in the culture supernatant of *L. brevis* SBC8803 responsible for HSP27 induction from Caco2/BBE cells, we first investigated the molecular weight of the HSP27-inducible molecule. The culture supernatant was separated by spin columns equipped with 5-, 10-, 30- or 50-kDa molecular weight cutoff (MWCO) membranes. HSP27 induction by filtrates passed through the 5- and 10-kDa MWCO membranes was diminished, but remained unchanged in filtrate passed through 30- and 50-kDa membranes (Figure 1C). These results indicated that the HSP27-inducible molecule had a relatively high molecular weight. In addition, the culture supernatant was precipitated by ammonium sulfate [(NH_4_)_2_SO_4_]. At 65% (NH_4_)_2_SO_4_ saturation, the precipitate strongly induced HSP27 (Figure 1D). This precipitate was further separated by diethylaminoethyl (DEAE) anion-exchange chromatography. The fraction eluted with 1.0 M sodium chloride strongly induced HSP27 (Figure 1E). This fraction was further separated by size-exclusion chromatography (Figure 1F). The HSP27-inducible fractions were collected and separated by high-pressure liquid chromatography (HPLC) and finally a single peak fraction was obtained (Figure 1G).This fraction significantly induced HSP27 (Figure 1H). Because HSP27 has been shown to inhibit cytochrome c-mediated activation of caspases [14], the inhibitory effect of this fraction was investigated using staurosporine-induced apoptosis in caco2/BBE cells. Caspase-9 and -3 activation were inhibited by this fraction (Figure 2).

**Figure 3:**
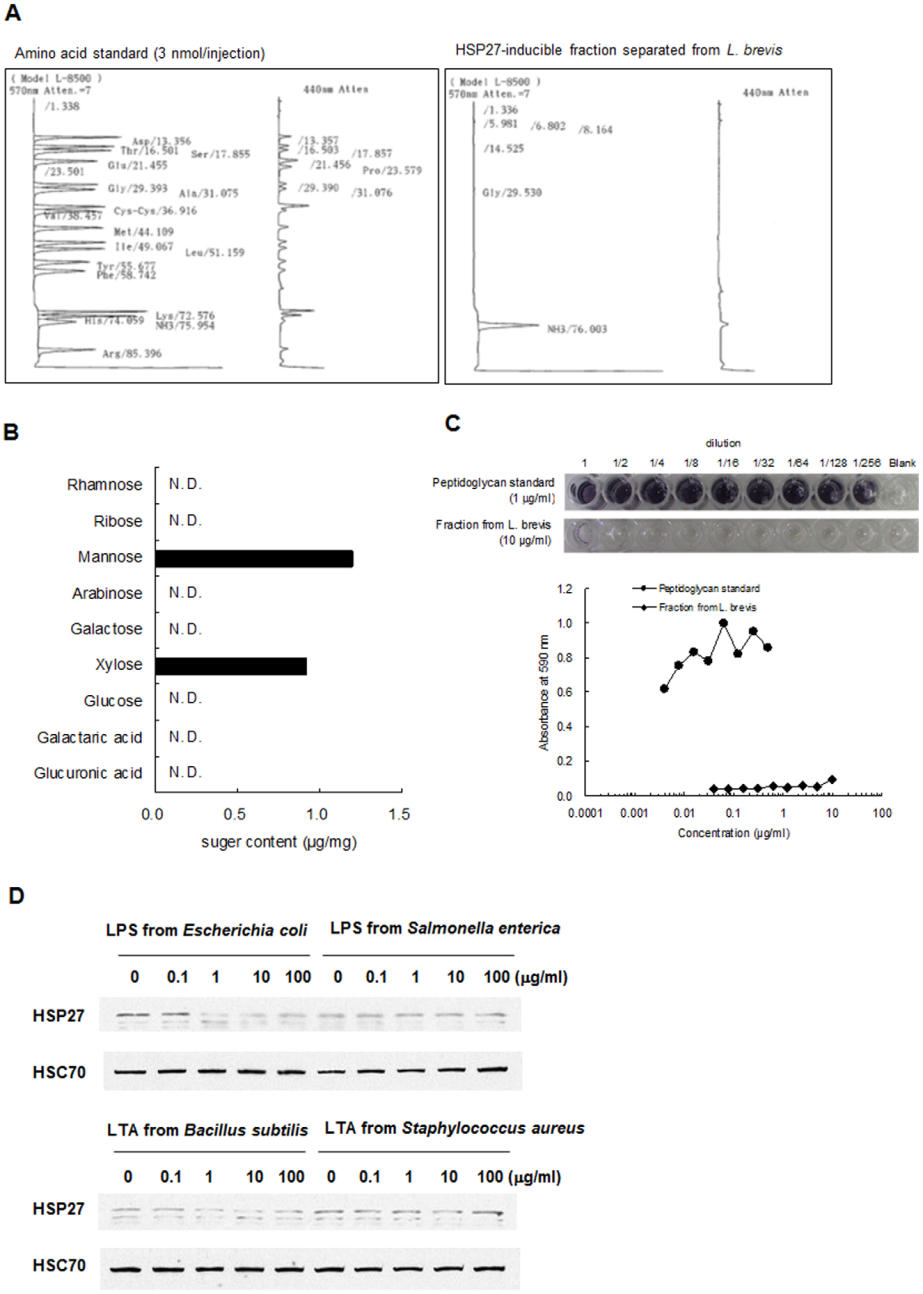
HSP27-inducible fraction separated from culture supernatant of *L. brevis* 8803 was neither protein nor polysaccharide. (A) Amino acid analysis after acid degradation of HSP27-inducible fraction by HPLC. (B) Neutral sugar and uronic acid content in the fraction. (C) Peptidoglycan content in the fraction. Concentration of peptidoglycan was measured by SLP test. (D) Western blot analysis of HSP27 induction from Caco2/BBE cells by LPS derived from *E.* coli or *Salmonella enterica*, or LTA derived from *B.* subtilis or *Staphylococcus aureus* (concentration: 0.1–100 µg/mL).

The molecular weight of this fraction was relatively high and had strong adsorption on the DEAE anion-exchange resin. Thus, we assumed that this fraction was protein and investigated the amino acid composition of this fraction by acid hydrolysis; however, no amino acids were detectable (Figure 3A). Furthermore, the total content of neutral sugar and uronic acid measured in the acid hydrolysate was <1% (Figure 3B) and the peptideglycan content determined by a silkworm larvae plasma test was extremely low (Figure 3C). Collectively, these results indicated that the HSP27-inducible fraction was neither a protein nor a polysaccharide. Bacterial cell wall lipopolysaccharide or lipoteichoic acid did not induce HSP27 from Caco2/BBE cells (Figure 3D). Unexpectedly, the purified material contained a large amount of phosphorus and oxygen; therefore, we measured the poly P contents by toluidine blue O (TBO) and molybdenum blue methods. The phosphoric acid content after acid hydrolysis was 0.92 mg/mg, (>90% w/w). The decrease in 620 nm absorbance of a 1 mg/mL solution caused by the metachromatic reaction with TBO was 0.097. Based upon these results, we assumed that the HSP27-inducible fraction was poly P.

**Figure 4:**
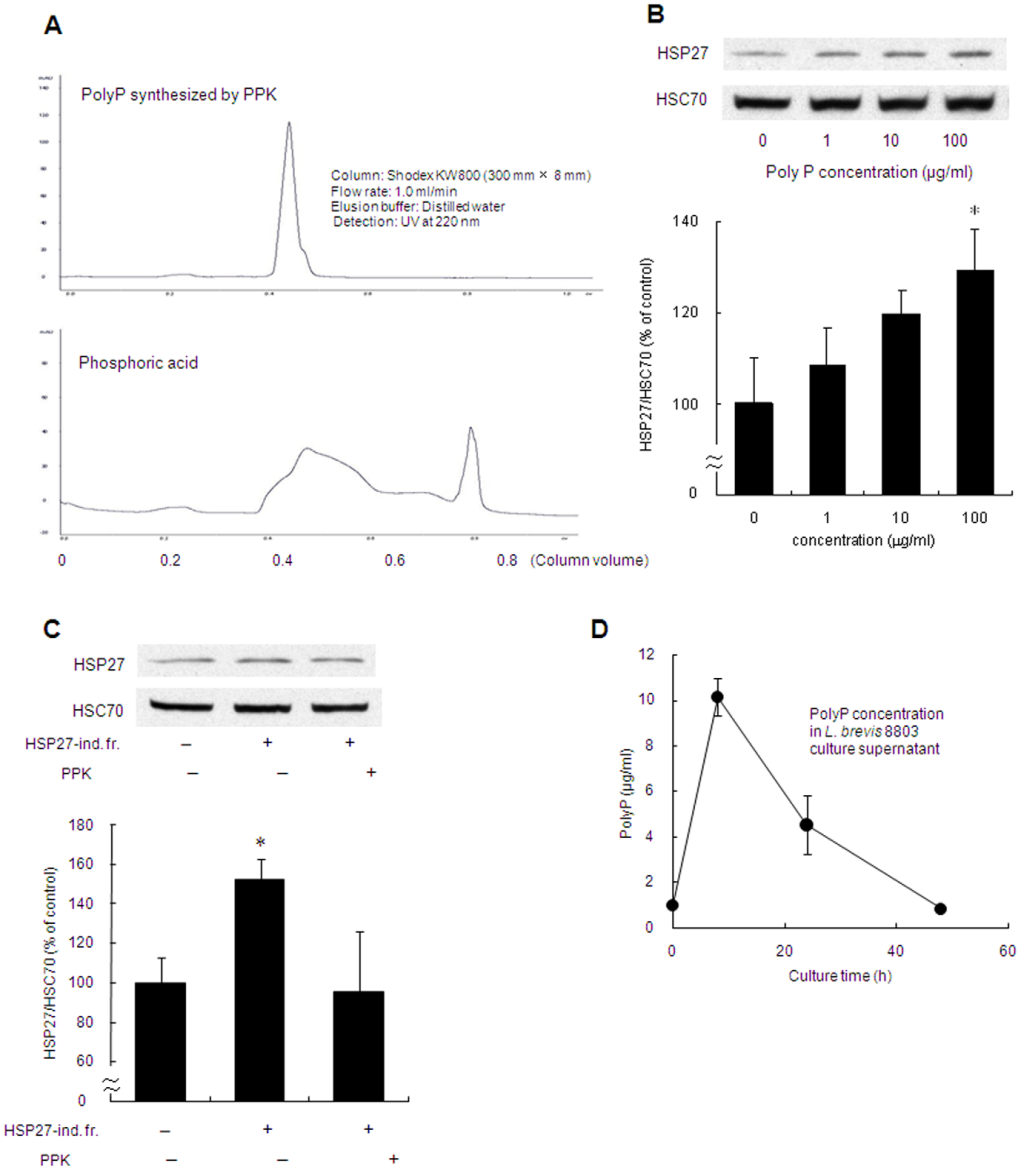
Enzymatically synthesized poly P induced HSP27 from Caco2/BBE cells through the activation of p38 MAPK pathway. (A) HPLC chromatogram of poly P synthesized by PPK. (B) Western blot analysis of HSP27 induction in Caco2/BBE cells by poly P synthesized by PPK (0–100 µg/mL), (n = 3). (C) Poly P degradation by PPK treatment with 10 µM ADP led to an inability to induce HSP27 from Caco2/BBE cells (n = 3). (D) Poly P content in the culture supernatant of *L. brevis* 8803 at 8, 24 and 24 h from bacterial culture (n = 3). * Significantly different from corresponding control group at *P*<0.05.

To confirm this supposition, poly P was synthesized by poly P-synthesizing enzyme polyphosphate kinase (PPK) and purified by HPLC (Figure 4A). The synthesized poly P significantly induced HSP27 from Caco2/BBE cells in a dose-dependent manner (Figure 4B). Furthermore, to confirm that poly P in the culture supernatant of *L. brevis* was responsible for HSP27 induction from Caco2/BBE cells, the ability to induce HSP27 by poly P was investigated when poly P in the HSP27-inducible fraction was degraded. Enzymatic reaction by PPK is reversible and usually favors poly P synthesis. If the reaction solution contains a large amount of adenosine diphosphate (ADP) in comparison to adenosine-5′-triphosphate (ATP), poly P is degraded to equilibrate the molecular ratio of ADP/ATP. After the treatment of PPK with a large amount of ADP (10 µmol/l), the HSP27-inducible fraction was unable to induce HSP27 production from Caco2/BBE cells (Figure 4C). This result suggests that poly P is the molecule responsible for induction of HSP27. Moreover, we confirmed that poly P content in the culture supernatant of lactobacilli varied in the culture time (Figure 4D).

### Improvement of the epithelial barrier function by Poly P through mediation of integrin–p38 MAPK pathway

**Figure 5:**
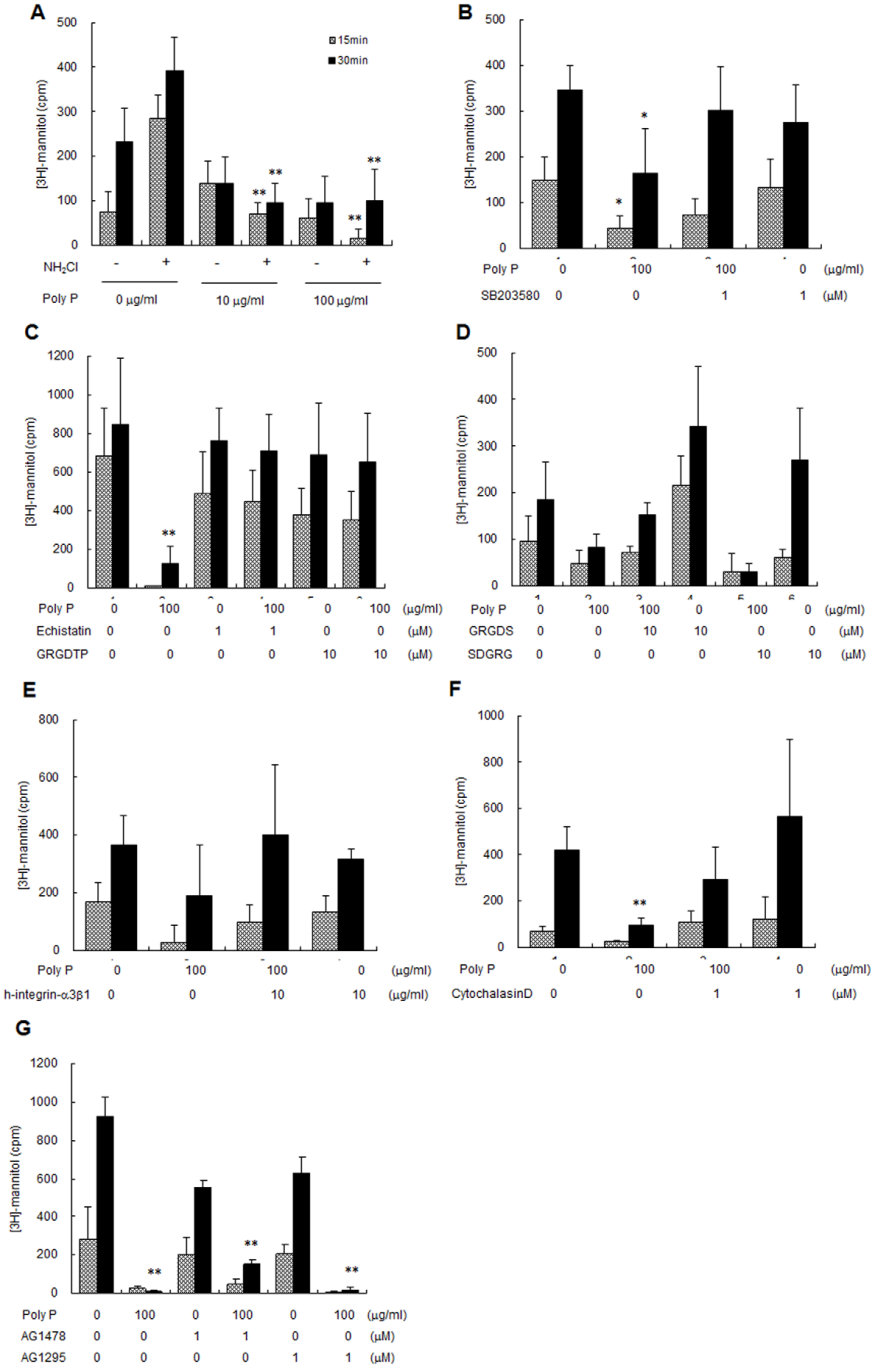
Poly P prevented [^3^H]-mannitol flux induced by exposure to an oxidant, and integrin and p38 antagonist inhibited its protective action. (A) 10 or 100 µg/mL of poly P inhibited transmural [^3^H]-mannitol fluxes in the presence or absence of 0.3 mM NH_2_Cl (n = 5, each group). (B–F) Inhibition of 0.3 mM NH_2_Cl-induced transmural [^3^H]-mannitol fluxes by 100 µg/mL of poly P was abolished by 1 µM of p38 MAPK inhibitor SB203580 (B), peptide antagonist of integrin, 1 µM of echistatin or 10 µM of Gly-Arg-Gly-Asp-Thr-Pro (GRGDTP) (C), 10 µM of reverse sequence of GRGDS, Ser-Thr-Asp-Gly-Arg-Gly (D), 1 µg/mL of human integrin α3β1 (E), 1 µM of cytochalasin D (F), 1 µM of EGFR inhibitor AG1478 or 1 µM of PDGFR inhibitor AG1296 treatment (n = 5, each group). *, ** Significantly different from corresponding control group at *P*<0.05 and *P*<0.01, respectively.

**Figure 6:**
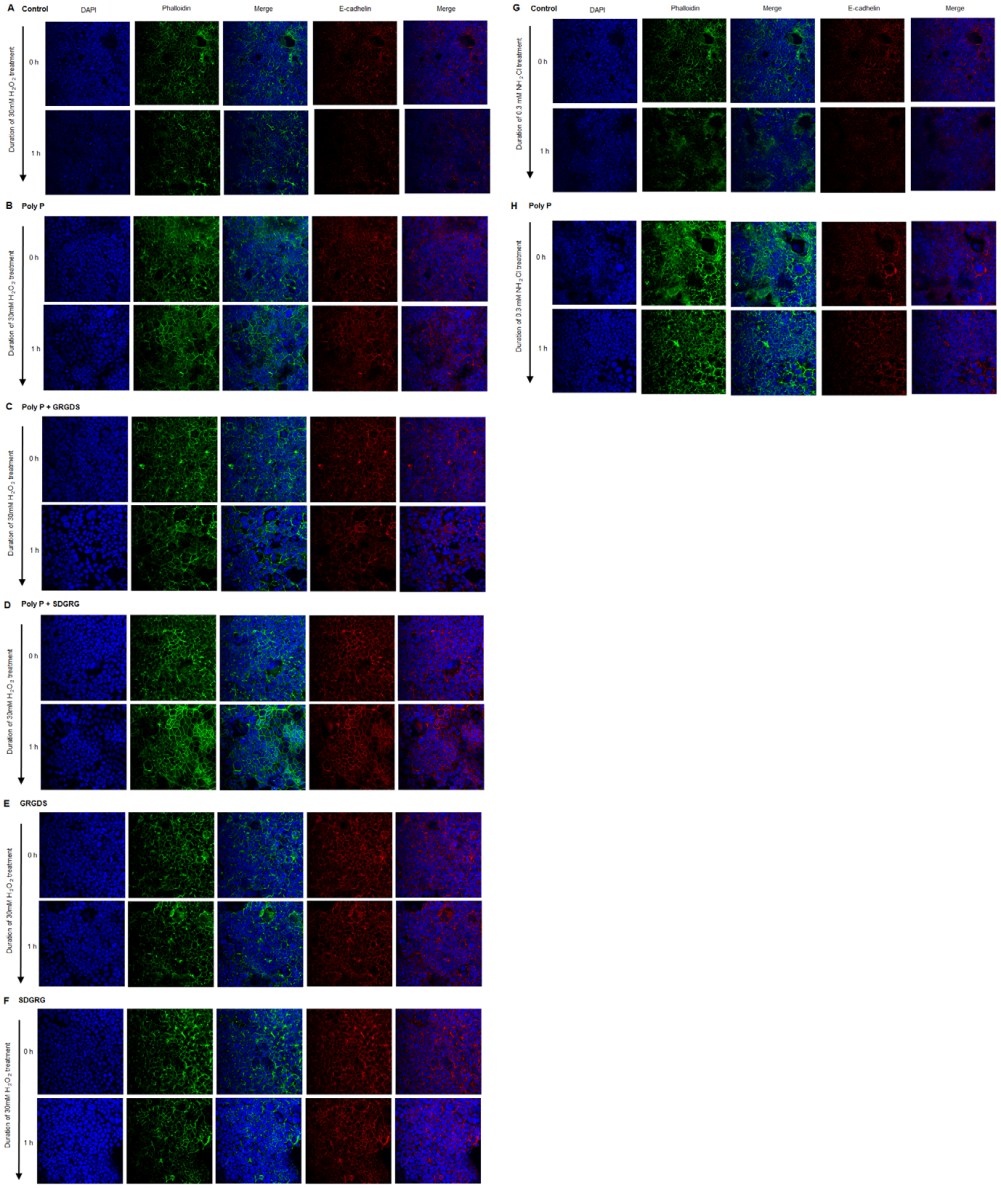
Poly P prevented F-actin and E-cadherin degradation induced by the oxidative stress. (A–F) Caco2/BBE cells were treated with 10 µg/mL of poly P, 10 µM of Gly-Arg-Gly-Asp-Ser (GRGDS) or Ser-Asp-Gly-Arg-Gly (SDGRG) overnight, followed by stimulated with 30 mM of H_2_O_2_ for 1 h. (G, H) Caco2/BBE cells were treated with 10 µg/mL of poly P overnight, followed by stimulated with 0.3 mM of NH_2_Cl for 1 h. F-actin was stained with phalloidin (green) and E-cadherin (red). Nuclei were counterstained with 4′,6-Diamidino-2-Phenylindole (DAPI) (blue).

To determine the physiologic consequences of poly P effects, transmural [^3^H]-mannitol fluxes were measured in intact small bowel loops to assess the intestinal barrier function. Increased mucosal permeability in small intestinal loops caused by exposure to an oxidant (NH_2_Cl, 0.3 mM) was significantly inhibited by 10 µg/mL of luminal poly P (Figure 5A). Phosphorylated HSP27 protects the actin cytoskeleton from depolymerization by interacting with actin [15], [16]. We confirmed that cytochalasin D, which severs actin filaments [17], inhibited the protective action by poly P (Figure 5F). Furthermore, poly P treatment of Caco2/BBE cells enhanced F-actin stability and attenuated its degradation induced by treatment with 30 mM hydrogen peroxide (Figures 6A, B), 0.3 mM of monochloramine (NH_2_Cl) (Figures 6G, H).

**Figure 7:**
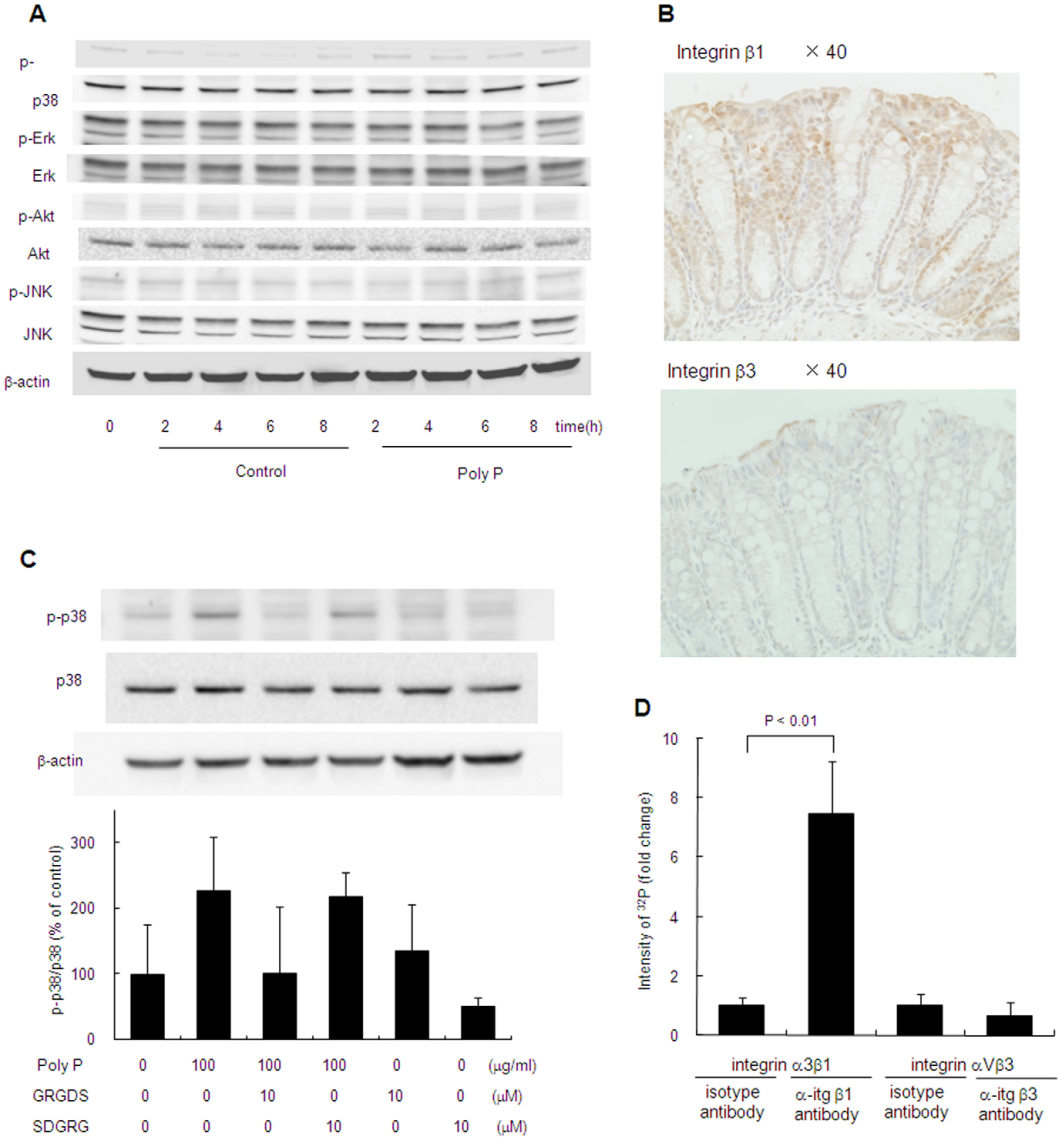
Poly P interacts with integrin β1 and activates p38 MAPK pathway. (A) Western blot analysis of p38, ERK, JNK and Akt phosphorylation in Caco2/BBE by 10 µg/mL of poly P. (B) Expression and localization of integrin β1 and β3 in mouse small intestine. Immunohistochemistry for integrin β1 and β3 were performed by using anti-integrin β1, rabbit-poly antibody and anti-integrin β3, rabbit-poly antibody. (C) Inhibition of poly P-induced p38 MAPK phosphorylation by RGD peptide. C57Bl/6 mice small intestinal loops were filled with RPMI 1640 medium containing 10 µg/mL of poly P, 10 µM of Gly-Arg-Gly-Asp-Ser (GRGDS) or Ser-Asp-Gly-Arg-Gly (SDGRG) 2 h at 37°C in a 5% CO_2_ incubator. Their p38 MAPK phosphorylation was detected by western blot (n = 3, each group). (D) Poly P-integrin β1 or β3 interaction analysis. Reaction mixture of ^32^P-labeled poly P and integrin α3β1 or αVβ3 was immunoprecipitated with integrin β1 or β3 antibody and protein G beads. Immunoprecipitated ^32^P-labeled poly P was determined with liquid scintillation counting.

To determine the signal transduction pathway for inducing HSP 27, the activation of the representative pathways including p38 MAPK, extracellular signal-regulated kinase (ERK), c-Jun N-terminal kinase (JNK) and Akt was examined. P38 MAPK was phosphorylated by poly P, while phosphorylation of ERK, JNK and Akt were not detected (Figure 7A). Then, the mediation of p38 MAPK to protect the intestinal epithelia against oxidant stress was investigated. The protective action against oxidant stress by poly P was inhibited by addition of the p38 MAPK inhibitor SB203580 (Figure 5B), indicating that the protective effect of poly P was mediated by activation of p38 MAPK.

Poly P appeared not to pass the tight junction of epithelial cells due to its high molecular weight. It was reported that integrins, which are a family of receptors that mediate cell adhesion to extracellular molecules, were associated with p38 MAPK [18]. We confirmed by immunohistochemical staining that integrin β1 and β3 molecules were apically expressed (Figure 7B). Peptide antagonists of integrin, echistatin, Gly-Arg-Gly-Asp-Thr-Pro (GRGDTP) and Gly-Arg-Gly-Asp-Thr-Ser (GRGDTS), which contain an Arg-Gly-Asp (RGD) sequence recognized by integrin ß [19], [20], completely inhibited the protective effect for the intestinal barrier function by poly P (Figures 5C, D). On the other hand, Ser-Thr-Asp-Gly-Arg-Gly (STDGRG), the reverse sequence of GRGDTS had no inhibitory activity, and integrin antagonists themselves did not increase the [^3^H]-mannitol flux induced by an oxidant (Figures 5D). Furthermore, the human integrin-α3β1 molecule inhibited the protective action by poly P (Figure 5E), indicating the almost all free poly P in the lumen was captured by the integrin molecule. P38 MAPK phosphorylation by poly P was inhibited by RGD peptide (Figure 7C). This poly P-induced F-actin stability against oxidative stress was reversed with the addition of the integrin antagonist GRGDS (Figures 6A–F). To confirm the ability of poly P on the binding with integrin β, we prepared a ^32^P-labeled poly P. Two µg/ml of a recombinant human integrin α3β1 or αVβ3 were incubated with ^32^P-labeled poly P for 1 hour at the room temperature. Integrins were then precipitated with anti-integrin β1 or β3 antibody. Collected protein G beads were washed three times with phosphate buffered saline (PBS), and then the radioactivity of immunoprecipitated ^32^P-labeled poly P was measured. The radioactivity of the sample precipitated with anti-integrin β1 antibody was significantly increased while that with anti-integrin β3 antibody was similar as the control. This demonstrates that poly P possess the ability to bind to integrin β1 (Figure 7D).

Growth factors have been known to protect the gastrointestinal epithelium from a variety of insults. Epidermal growth factor (EGF) prevents acetaldehyde-induced disruption of tight junctions in the Caco-2 cell monolayer by PLCγ-dependent protein kinase C activation [21]. Moreover, it has been reported that poly P activated the mitogenic action of growth factors such as fibroblast growth factor by enhancing their physical interactions [22]. However, in our study, neither EGF receptor inhibitor AG1478 (1 µM) nor platelet-derived growth factor receptor inhibitor AG1296 (1 µM) suppressed the protective action by poly P (Figure 5G).

Collectively, integrin–p38 MAPK signaling pathway is mediated in the actions of poly P, which is responsible for protecting the intestinal epithelia.

### Poly P improved intestinal injuries and survival rate of mice treated with a lethal dose of dextrin sodium sulfate (DSS)

**Figure 8:**
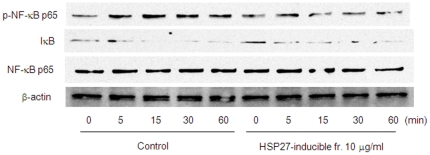
Anti-inflammatory property of HSP27-inducible fraction. Time course of NF-κB pathway activation in Caco2/BBE cells induced by 10 ng/mL TNF-α treatment in the presence or absence of 10 µg/mL of poly P.

**Figure 9:**
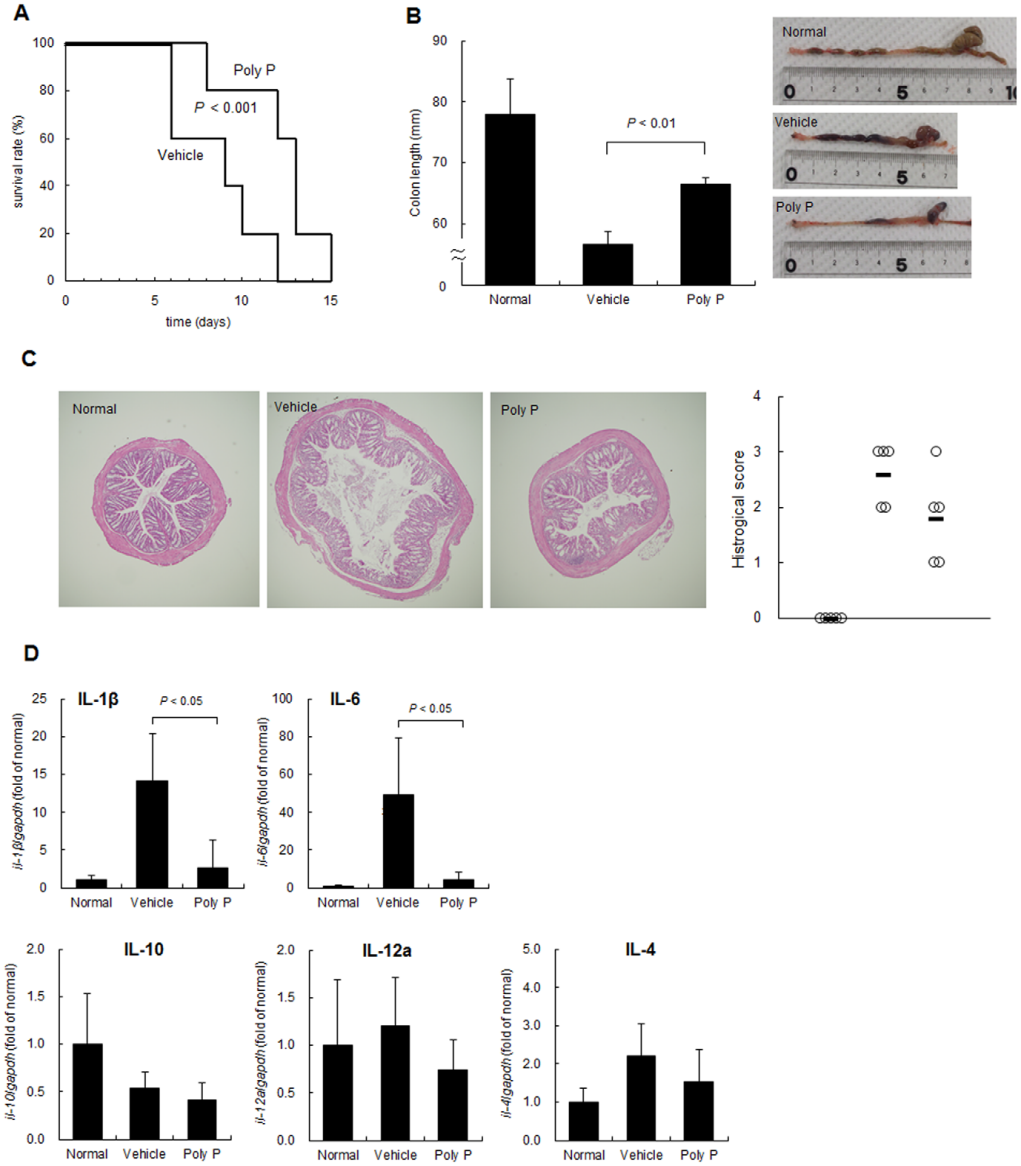
Poly P improved intestinal injury and survival rate of mice treated with a lethal dose of DSS. (A) Survival of 4% DSS administered C57Bl/6 mice challenged intrarectally with 10 µg/100 µL of poly P or 100 µL of PBS once a day throughout the experimental period (n = 5, each group). (B–D) Representative picture of a colon and average colon length (B), representative colon histopathology and histological score (C) and cytokines, IL-1β, IL-6, IL-10, IL-12a and IL-4 expression in the colon (D) of C57Bl/6 mice, which were orally administered 0% (normal) or 3% DSS, challenged intrarectally with 10 µg/100 µL of poly P or 100 µL of PBS (vehicle) once a day for 7 days (n = 5, each group).

HSP27 has been shown to have an anti-inflammatory property [23]. After we confirmed TNF-α–induced phosphorylation of NF-κB and degradation of IκB was inhibited by HSP27-inducible fraction (Figure 8), the effect of poly P on a DSS-enteritis model, in which the barrier function of the intestinal epithelia was injured and led to increased exposure to luminal antigen [24], was analyzed. C57Bl/6 mice were orally administered 4% DSS and intrarectally administered 10 µg of poly P every day throughout the experimental period. Poly P-treated mice survived longer compared to PBS-treated control mice (Figure 9A). The cumulative survival rate of the poly P-treated mice was significantly higher than that of PBS-treated mice. In addition, C57Bl/6 mice were orally administered 3% DSS, and intrarectally administered 0 or 10 µg of poly P every day for seven days, after which the mice were euthanized. The mice that were intrarectally administered 10 µg of poly P did not show any significant colon shrinkage compared to normal mice, although the colon length in PBS-treated mice was significantly decreased by oral administration of 3% DSS (Figure 9B). Hematoxylin-eosin (H&E) staining of the colonic sections showed that poly P-treated mice experienced improvements in intestinal injuries compared to PBS-treated mice (Figure 9C). The proinflammatory cytokines IL-1β and IL-6 have been reported to be upregulated in the acute DSS colitis model [25], [26]. Treatment with 3% DSS for seven days caused a significant increase in the proinflammatory cytokines IL-1β and IL-6 in mice colons compared to those of mice who were administered distilled water. On the other hand, intrarectal administration of 10 µg of poly P prevented DSS-induced proinflammatory cytokine upregulation (Figure 9D).

## Discussion

This study, for the first time, demonstrated that poly P, a common product of lactobacilli and other bacteria associated with intestinal microflora, is responsible for probiotic actions that protect the intestinal epithelia from oxidant stress and improve epithelial injury due to excess inflammation. It is noteworthy that this probiotic action is required to activate the integrin–p38 MAPK pathway, which is a novel system for mediating host–bacterial interactions. Inorganic poly P is a polymer composed of tens to hundreds of phosphate residues linked by phosphoanhydride bonds. Poly P is ubiquitous in nature and widely distributed among microorganisms [27]. PPK plays an important role in poly P metabolism in bacteria. This enzyme reversibly catalyzes the polymerization of the terminal phosphate of ATP into a poly P chain [28]. Our study suggests that poly P secreted from many probiotic and commensal bacteria mediates the maintenance of intestinal homeostasis because they carry PPK and the poly P-degrading exopolyphosphatase gene in their genome.

This study demonstrated that the protective effect of poly P on intestinal epithelia was eliminated by inhibitors of integrins or p38 MAPK (Figure 5). In addition, p38 MAPK phosphorylation by poly P was also inhibited by the integrin inhibitor (Figure 7C). Furthermore, we confirmed that integrin β1 and β3 molecules were apically expressed in mouse small intestine (Figure 7B), as previously reported [29], [30], and poly P specifically binds to integrin β1 (Figure 7D). These results suggest that poly P develops a robust intestinal barrier function through interaction with integrin β1, followed by the p38 pathway activation. Integrins are obligate heterodimers containing two distinct chains called α and β subunits [31], which mediate attachment between cytoskeletal components, in particular the microfilaments inside the cell and ECM. They also play a role in cell signaling, defining cellular shape and mobility, and regulating the cell cycle. However, the role of integrins in host–microbial interactions had been unknown. While functional molecules produced by bacteria which mediate host–microbial interactions are recognized by toll-like receptors [32], [33] or transporters [7], [34], the present study is the first to propose that integrins are novel sensors for the probiotic-released molecules contributing to the beneficial function of probiotics.

P38 MAPK is a class of mitogen-activated protein kinases responsive to stress stimuli, such as cytokines, ultraviolet irradiation, heat shock, and osmotic shock, and is involved in cell differentiation and apoptosis. It has been reported that an intraperitoneal injection of the p38 MAPK-specific inhibitor FR167653 aggravated 3% DSS-induced colitis [35]. This suggests that activation of the p38 MAPK pathway is a protective reaction against the excess inflammation. Other strains of *lactobacillus* including LGG also stimulated the phosphorylation of p38 MAPK [36], thus suggesting that the activation of p38 MAPK is a pivotal process in the exhibition of the beneficial functions of such bacterial strains.

We also confirmed that poly P content secreted from the bacterium varied in the culture time (Figure 4D). In addition, positive relationship between secreted poly P content and HSP27 induction by the culture supernatant was observed. *L. brevis* began to accumulate poly P during its exponential growth phase and reached a maximum when the cells were in the early stationary phase. Similarly, it has been reported that poly P was accumulated during the growth phase in the case of *Escherichia coli* [37], suggesting that secretion and accumulation of poly P is dependent on intestinal condition. In unsuitable conditions due to the propagation of pathogenic bacteria or administration of antibiotics, the ability to produce or secrete poly P by probiotics and commensal bacteria is thought to decrease, thus leading to failed maintenance of intestinal homeostasis and the development of intestinal disorders. It is also probable that the probiotic effect on intestinal disorders is not stable when live bacteria are used. The administration of poly P rather than live bacteria is expected to exert beneficial functions regardless of the condition of the gastrointestinal tract. Moreover, unraveling the mechanism of poly P accumulation and secretion by intestinal bacteria may lead to the development of novel strategies to prevent and/or treat intestinal disorders.

In a DSS-induced acute experimental colitis model, intrarectal administration of poly P significantly improved the survival rate and ameliorated intestinal injury (Figure 9A–C). Intrarectal administration of poly P also suppressed the production of inflammatory cytokines IL-1β and IL-6 in mice colons with DSS-induced colitis (Figure 9D). In our *ex vivo* loop assay, poly P improved the intestinal barrier function (Figure 5A). Furthermore, staurosporine-induced caspase-9 and -3 activation and TNF-α-induced NF-κB pathway activation were inhibited by pretreatment with the HSP27-inducible fraction (Figures 2 and 8). Accordingly, both the cytoprotective and anti-inflammatory properties of poly P probably contribute to improve the intestinal injury caused by DSS-induced colitis.

It has been reported that poly P is essential for the survival of pathogenic bacteria such as *Salmonella*, *Shigella*, *Pseudomona aeruginosa*and, and *Neisseria meningitidis*, which produce virulent factors [38]–[41]. However, it is unclear whether poly P itself is a virulent factor. In our *ex vivo* loop study and DSS-induced mouse colitis model, poly P showed beneficial functions without any adverse effects. However, it remains unclear whether pathogenic bacteria-derived poly P is secreted into its living environment, while some probiotic bacteria are thought to secrete poly P in the culture supernatant as shown in this study. Therefore, poly P produced by pathogens appears to be used as an intracellular energy source, whereas probiotic-derived poly P can be released and can exert multiple functions including a cytoprotective effect. Further analysis of the mechanism by which probiotics secrete poly P from the cytoplasm to the extracellular environment is needed to discriminate the functions of probiotic-derived poly P from those of pathogen-derived poly P.

In summary, the present study showed that poly P produced by probiotics was a bioactive molecule that induced cytoprotective HSP through activation of the integrin–p38 MAPK pathway, and prevented oxidant-induced intestinal barrier weakening. The integrin–p38 MAPK pathway is a novel system for sensing the bioactive components secreted by probiotics and commensal bacteria. Furthermore, poly P ameliorated epithelial injury in the colon of mice with DSS-induced colitis, illustrating that poly P is a promising agent for preventing and alleviating intestinal inflammation. Determining the secretory mechanism of poly P by probiotic and/or commensal bacteria essential for maintaining intestinal homeostasis will lead to a better understanding of the pathogenesis of inflammatory disorders and aid future therapeutic development.

## Materials and Methods

### Microorganisms

*L. brevis* SBC8803 and SBC8013 used in this study was supplied by Sapporo Breweries Ltd. LGG was purchased from the American Tissue Culture Collection (ATCC). These lactobacilli were cultured in de Man–Rogosa–Sharpe (MRS) broth (Difco Laboratories, Detroit, MN) for a day at 37°C.

### Cell culture

Human colonic epithelial Caco2/BBE cells were purchased from ATCC. They were grown in high-glucose Dulbecco's Modified Eagle's medium supplemented with 10% (vol/vol) fetal bovine serum (FBS), 2 mM L-glutamine, 50 U/mL penicillin, 50 µg/mL streptomycin and 10 µg/mL transferrin (all from Invitrogen/GIBCO, Grand Island, NY) in a humidified atmosphere of 5% CO_2_. The cells were plated on 6- or 12-well plates at a density of 10^5^ cells/cm^2^ and then allowed to differentiate for 10–14 days before experiments.

### Mice

The studies were approved by the Institutional Animal Care and Use Committee of the Asahikawa Medical University (the permit number 10080). C57Bl/6 mice were purchased from Sankyo Labo Service (Tokyo,Japan).

### Isolation of HSP27-inducible substance from *L. brevis* SBC8803 culture supernatant

The culture medium was centrifuged at 500× *g* for 10 min to obtain the culture supernatant, which was then filtered through a 0.2-µm membrane. While stirring, ammonium sulfate was added to the supernatant to give 65% saturation, and then the mixture was centrifuged at 5000× *g* for 10 min. Further, ammonium sulfate was added to the obtained supernatant to give 90% saturation, and then the mixture was centrifuged at 5000× *g* for 10 min. The precipitates were redissolved in distilled water, and then desalted using a dialysis tube equipped with molecular weight 7000 cutoff membrane (SnakeSkin dialysis tube, Thermo Scientific, Rockford, IL). The ammonium sulfate precipitate was separated by anion-exchange chromatography. The sample was applied to a DEAE Sephadex A-50 column (GE Healthcare, Buckinghamshire, UK). The column was eluted with 0 M, 0.1 M, 0.5 M, and 1.0 M NaCl dissolved in 20 mM Tris-HCl (pH 8.5) stepwise, and each fraction was collected. Next the HSP27-inducible fraction obtained from the DEAE anion-exchange chromatography was separated by size-exclusion chromatography. The sample was applied to a Sepahdex G-100 (GE Healthcare), and eluted with 20 mM phosphate buffer (pH 6.5). Finally, the sample was separated using an ÄKTA design HPLC system (GE Healthcare) using a Shodex KW800 column (300 mm×8 mm ID, Showa denko, Tokyo, Japan). The sample solution was eluted with 20 mM phosphate buffer (pH 6.5) at a flow rate of 0.1 ml/min. The eluent was monitored with an ultraviolet spectrophotometer at 220 nm.

### Quantification of poly P

Polyphosphate was quantified by metachromatic assay with the TBO method, and phosphoric acid was quantified by the Mo-blue method after acid hydrolysis of Poly P.

The TBO method was based on the decrease in absorbance at 620 nm by the metachromatic reaction of TBO with poly P. Ten microliters of sample solution was mixed with 500 µL of TBO assay solution (15 mg/mL TBO and 0.1 N acetic acid) and left for 15 min at room temperature. The absorbance at 620 nm was measured within 30 min.

For the Mo-blue method, 5 µL of sample solution was mixed with an equal volume of 2 N HCl and heated at 95°C for 30 min for acid degradation of poly P. The solution was then diluted to 300 µL with distilled water, mixed with 700 µL of Mo-blue assay solution (0.42% (NH_4_)_6_Mo_7_O_24_ 4H_2_O dissolved in 1 N H_2_SO_4_∶10% ascorbic acid = 6∶1), and incubated at 45°C for 20 min. Phosphoric acid concentration was determined from the absorbance at 750 nm and calculated from a standard phosphoric acid solution curve.

### Synthesis and degradation of poly P by PPK

To synthesize poly P, 1 mL of reaction mixture containing 50 mM Tris-HCl (pH 7.4), 40 mM ammonium sulfate, 4 mM MgCl_2_, 40 mM creatine phosphate, 20 ng/mL creatine kinase, 1 mM ATP (pH 7.2), and 1 U of polyphosphate kinase (PPK) from *Propionibacterium shermanii* was incubated for 3 h at 37°C. To purify enzymatically synthesized poly P from the reaction mixture, 100 µL of 50 mM CaCl_2_ was added to the reaction mixture to aggregate the synthesized poly P and then centrifuged at 5000× *g* for 10 min. The precipitates were dissolved by adding 50 mM ethylenediaminetetraacetic acid (EDTA) solution, and then low-molecular weight components such as Ca^2+^ ions and EDTA were removed from the solution by dialysis using a tube equipped with 3-kDa MWCO membrane.

To degrade poly P, 2 µL of poly P solution was incubated at 37°C for 3 h in the final reaction mixture (50 mmol/L (NH_4_)_2_SO_4_, 4 mmol/L MgCl_2_, 10 µmol/L ADP, 40 mmol/L HEPES-KOH [pH 7.5], and 1 U/µL PPK).

### Western blots

After washing Caco2/BBE cells with PBS, proteins were extracted from samples using a Mammalian Cell Extraction Kit (BioVision, Mountain View, CA) and analyzed by Western blotting. Ten to thirty micrograms proteins of each sample was resolved by SDS-PAGE (12.5%) and immediately transferred to a nitrocellulose membrane using a transfer buffer (25 mM Tris, pH 8.8, 192 mM glycine with 20% (vol/vol) methanol). Nitrocellulose membranes were incubated in PBS with 0.05% (vol/vol) Tween 20 (T-PBS) containing 5% (wt/vol) skim milk or 1% (wt/vol) bovine serum albumin (BSA, Sigma-Aldrich, St. Louis, MO) for 1 h at room temperature to block nonspecific binding. The blot was incubated overnight at 4°C with HSP27, HSC70 antibody (Stressgen, Victoria, British Columbia, Canada), anti-human p38 MAPK and phospho-p38 MAPK, anti-human extracellular signal-regulated kinase (ERK) and phospho-ERK, anti-human c-Jun N-terminal kinase (JNK), phospho-JNK, anti-human phosphatidylinositol 3-kinase (Akt), phospho-Akt, anti-human NF-κB p65, phospho-NF-κB p65, IκB (Cell Signaling Technology, Danvers, MA) or anti-human β-actin (Imgenex, San Diego, CA) as the primary antibody. Blot was washed 3 times for 10 min each in T-PBS at room temperature, incubated for 60 min in species-appropriate horseradish peroxidase-conjugated secondary antibody (R&D systems, Minneapolis, MN) in T-PBS, washed 3 times in T-PBS, and developed using the Super-Signal West Pico enhanced chemiluminescence system (Pierce Chemical, Rockford, IL).

### Real-time RT-PCR

Total RNA was extracted from frozen mouse colon using Trizol (Invitrogen), and purified with an RNeasy mini kit (Qiagen, Tokyo, Japan) according to the manufacturer's instructions. The quality and quantity of total RNA was verified by agarose gel electrophoresis and spectrophotometry. Genomic DNA was eliminated, and single-stranded complementary DNA (cDNA) was synthesized using a QuantiTect reverse transcription kit (Qiagen). Gene expression was measured by real-time RT-PCR using a LightCycler 480 (Roche Applied Science) using specific primers (sense, 5′-tccaggatgaggacatgagcac-3′, anti-sense, 5′-gaacgtcacacaccagcaggtta-3′ for the analysis of mouse IL-1β, sense, 5′-aagccagagctgtgcagatgagta-3′, anti-sense, 5′-tgtcctgcagccactggttc-3′ for the analysis of mouse IL-6, sense, 5′-gaccagctggacaacatactgctaa-3′, anti-sense, 5′-gataaggcttggcaacccaagtaa-3′ for the analysis of mouse IL-10, sense, 5′-tgtcttagccagtcccgaaacc-3′, anti-sense, 5′-tcttcatgatcgatgtcttcagcag-3′ for the analysis of mouse IL-12, and sense, 5′-tctcgaatgtaccaggagccatatc-3′, anti-sense,5′-agcaccttggaagccctacaga-3′ for the analysis of mouse IL-4) in triplicate. The averaged mRNA expression of these cytokines was normalized to GAPDH expression (sense, 5′-tgtgtccgtcgtggatctga-3′, anti-sense, 5′-ttgctgttgaagtcgcaggag -3′ for the analysis of mouse GAPDH,). Serial dilutions of a standard solution were included for each gene to generate a standard curve and allow calculation of the input amount of cDNA for each gene. A LightCycler 480 optical capillary tube was incubated at 95°C for 10 min to activate the FastStart Taq DNA polymerase. The run condition were 45 cycles at 95°C for 10 s, 59°C for 20 s, and 72°C for 50 s. Melting curves of each amplified gene were created to obtain PCR efficiency.

### Ex vivo intestinal loop studies

C57Bl/6 mice were sacrificed and the small intestine was removed beginning at the ligament of Treitz. The small intestine was divided into 3–6 pieces, each end ligated with silk sutures and the loops filled with RPMI 1640 medium containing sample. Loops were incubated for 2 h at 37°C in a 5% CO_2_ incubator. To measure permeability effects, the loops were filled with RPMI 1640 medium containing 1 µCi/mL [^3^H]-mannitol with or without 0.3 mM freshly prepared monochloramine (NH_2_Cl). Loops were placed into the middle section of the organ culture dish in 4 mL of RPMI 1640 without NH_2_Cl. RPMI 1640 mediums in the culture dish were taken at 5, 20 and 35 min to determine the flux of mannitol from the lumen to the medium outside bathing loops that were being bathed.

### Induction and assessment of colitis

C57Bl/6 mice were administered 3% (wt/vol) DSS (MW, 2500 Da) in the drinking water. In the test group (n = 5), 10 µg of *L. brevis*-derived poly P dissolved in 100 µL of PBS was intrarectally administered once a day throughout the experimental period. In the control group (n = 5), 100 µL of PBS was intrarectally administered once a day throughout the experimental period. The mice were sacrificed on the 7th day following DSS treatment and the entire colon was removed from the cecum to the anus, and the colon length was measured as a marker of inflammation. Gene expression in the colon was investigated using real-time RT-PCR. In another experiment, the survival rate of 4% DSS-administered mice was investigated.

### Poly P-integrin interaction assay

To synthesize ^32^P-labeled poly P, 1 mL of reaction mixture containing 50 mM Tris-HCl (pH 7.4), 40 mM ammonium sulfate, 4 mM MgCl_2_, 40 mM creatine phosphate, 20 ng/mL creatine kinase, 1 mM ATP (pH 7.2), 100 µCi of [γ-^32^P]ATP and 1 U of polyphosphate kinase (PPK) from *Propionibacterium shermanii* was incubated for 3 h at 37°C, and the reaction was stopped by adding 100 µL of 50 mM CaCl_2_ and then centrifuged at 5000×g for 10 min. The precipitates were dissolved by adding 50 mM ethylenediaminetetraacetic acid (EDTA) solution, and then low-molecular weight components such as Ca^2+^ ions and EDTA were removed from the solution by dialysis using a tube equipped with 3-kDa MWCO membrane. Twenty µl of 100 µg/ml human integrin α3β1 or αVβ3 was added into 50 µl of ^32^P-labeled poly P solution and reacted for 1 h at room temperature. Reaction mixtures were immunoprecipitated with 1 µg of anti-human integrin β1 antibody, mouse IgG1 isotype control antibody, anti-human integrin β3 antibody, or goat IgG isotype control antibody (R&D systems) and 20 µl of Bio-Adembeads Protein G beads (Ademtech, Pessac France). After 1 h incubation at room temperature, immunoprecipitated ^32^P-labeled poly P was determined by liquid scintillation counting.

### Histological evaluation of colitis

The colons were fixed in 10%-buffered formalin solution, embedded in paraffin using standard methods, cut into 5-µm sections, stained with H&E, and then assessed under light microscopy. The histological activity was assessed according to Berg's score described below [42]. The grades of the intestinal inflammation were assessed in three representative parts of the colon in each mouse because of multi focal and variable severities of the intestinal lesions. A score from 0 to 4 was based on the following criteria: (grade 0) no change from normal tissue; (grade 1) one or a few multi focal mononuclear cell infiltrates in the lamina propria accompanied by minimal epithelial hyperplasia and slight to no depletion of mucus from goblet cells; (grade 2) the lesions tended to involve more of the intestine than grade 1 lesions, or were more frequent. Typical changes included mild inflammatory cell infiltrates in the lamina propria composed primarily of mononuclear cells with a few neutrophils. Small epithelial erosions were occasionally present and inflammation rarely involved the submucosa; (grade 3) lesions involved a large area of mucosa or were more frequent than grade 2 lesions. Inflammation was moderate and often involved the submucosa but was rarely transmural. Inflammatory cells were a mixture of mononuclear cells as well as neutrophils, and crypt abscesses were sometimes observed. Ulcers were occasionally observed; (grade 4) such lesions usually involved most of the intestinal section and were more severe than grade 3 lesions. Inflammation was severe, including mononuclear cells and neutrophils, and it was sometimes transmural. Crypt abscesses and ulcers were present.

### Immunohistochemistry

Immunohistochemistry for integrin β1 and β3 were performed by using anti-integrin β1, rabbit-poly (I782, Bioworld Technology, Louis Park, MN) and anti-integrin β3, rabbit-poly (Ab-773, Enogene, New York, NY). After deparaffinization and rehydration, endogenous peroxidase activity was blocked with 0.6% H_2_O_2_ in methanol for 25 min. The slides were then treated with the antigen-retrieval technique based on microwave oven heating in 10 mM citrate buffer (pH 6.0) for 20 min. The container was allowed to cool at room temperature for 20 minutes. After blocking any nonspecific reaction with 10% goat serum in PBS, sections were incubated with primary antibody at 4°C overnight. This step was followed by sequential incubation with biotin-labeled goat anti-rabbit IgG and avidin biotin complex reagents (Vector Laboratories, Burlingame, CA). The biotinylated second antibody diluted at 1∶100 for 30 min at room temperature. Sections were visualized with diaminobenzidine-H_2_O_2_ solution, and counterstained with haematoxylin.

### Confocal immunofluorescence microscopy

Cells were plated on chamber slides and allowed to culture for 1–2 weeks. Slides were fixed for 15 min in 4% paraformaldehyde, washed extensively with PBS, permeabilized with 0.1% Triton X-100 for 15 min and blocked in 3% BSA in PBS for 1 h at room temperature. Slides were then sequentially incubated with primary antibodies E-cadherin (BD transduction Laboratories) and Alexa 488-conjugated phalloidin (Lonza) for 1 h at room temperature, washed with PBS, and then incubated with Alexa 594-conjugated secondary antibody (Invitrogen-Molecular Probes) for 1 h. The nuclei were counterstained with DAPI (Lonza) for 5 min. Cells were mounted with an anti-fade mounting medium, and immunofluorescence was visualized with a confocal microscope.

### Statistical analysis

All values were expressed as the standard error of the mean (SEM). Statistical evaluation of the results was performed by one-way analysis of variance (ANOVA) followed by the Fisher's least significant difference (LSD). Statistical evaluation for the survival rate difference was performed by the log-rank test. A probability value of less than 0.05 was considered statistically significant.

## References

[1] Hooper LV, Gordon JI (2001). Commensal host-bacterial relationships in the gut.. Science.

[2] De Keersmaecker SC, Verhoeven TL, Desair J, Marchal K, Vanderleyden J (2006). Strong antimicrobial activity of *Lactobacillus rhamnosus* GG against *Salmonella typhimurium* is due to accumulation of lactic acid.. FEMS Microbiol Lett.

[3] Ayabe T, Satchell DP, Wilson CL, Parks WC, Selsted ME (2000). Secretion of microbicidal α-defensins by intestinal Paneth cells in response to bacteria.. Nat Immunol.

[4] Vaishnava S, Behrendt CL, Ismail AS, Eckmann L, Hooper LV (2008). Paneth cells directly sense gut commensals and maintain homeostasis at the intestinal host-microbial interface.. Proc Natl Acad Sci USA.

[5] Belkaid Y, Oldenhove G (2008). Tuning microenvironments: induction of regulatory T cells by dendritic cells.. Immunity.

[6] Zyrek AA, Cichon C, Helms S, Enders C, Sonnenborn U (2007). Molecular mechanisms underlying the probiotic effects of *Escherichia coli* Nissle 1917 involve ZO-2 and PKCξ redistribution resulting in tight junction and epithelial barrier repair.. Cell Microbiol.

[7] Fujiya M, Musch MW, Nakagawa Y, Hu S, Alverdy J (2007). The *Bacillus subtilis* quorum-sensing molecule CSF contributes to intestinal homeostasis via OCTN2, a host cell membrane transporter.. Cell Host Microbe.

[8] Yan F, Cao H, Cover TL, Whitehead R, Washington MK (2007). Soluble proteins produced by probiotic bacteria regulate intestinal epithelial cell survival and growth.. Gastroenterology.

[9] Ménard S, Candalh C, Bambou JC, Terpend K, Cerf-Bensussan N (2004). Lactic acid bacteria secrete metabolites retaining anti-inflammatory properties after intestinal transport.. Gut.

[10] Ewaschuk JB, Diaz H, Meddings L, Diederichs B, Dmytrash A (2008). Secreted bioactive factors from *Bifidobacterium infantis* enhance epithelial cell barrier function.. Am J Physiol Gastrointest Liver Physiol.

[11] Petrof EO, Claud EC, Sun J, Abramova T, Guo Y (2009). Bacteria-free solution derived from *Lactobacillus plantarum* inhibits multiple NF-κB pathways and inhibits proteasome function.. Inflamm Bowel Dis.

[12] Heuvelin E, Lebreton C, Grangette C, Pot B, Cerf-Bensussan N (2009). Mechanisms involved in alleviation of intestinal inflammation by bifidobacterium breve soluble factors.. PLoS One.

[13] Ueno N, Fujiya M, Segawa S, Nata T, Moriichi K (2011). Heat-killed body of lactobacillus brevis SBC8803 ameliorates intestinal injury in a murine model of colitis by enhancing the intestinal barrier function.. Inflamm Bowel Dis.

[14] Bruey JM, Ducasse C, Bonniaud P, Ravagnan L, Susin SA (2000). Hsp27 negatively regulates cell death by interacting with cytochrome c.. Nat Cell Biol.

[15] Guay J, Lambert H, Gingras-Breton G, Lavoie JN, Huot J (1997). Regulation of actin filament dynamics by p38 map kinase-mediated phosphorylation of heat shock protein 27.. J Cell Sci.

[16] Garcia MC, Ray DM, Lackford B, Rubino M, Olden K (2009). Arachidonic acid stimulates cell adhesion through a novel p38 MAPK-RhoA signaling pathway that involves heat shock protein 27.. J Biol Chem.

[17] Turner JR (2006). Molecular basis of epithelial barrier regulation: from basic mechanisms to clinical application.. Am J Pathol.

[18] Ivaska J, Reunanen H, Westermarck J, Koivisto L, Kähäri VM (1999). Integrin α2β1 mediates isoform-specific activation of p38 and upregulation of collagen gene transcription by a mechanism involving the α2 cytoplasmic tail.. J Cell Biol.

[19] Kumar CC, Nie H, Rogers CP, Malkowski M, Maxwell E (1997). Biochemical characterization of the binding of echistatin to integrin αβ3 receptor.. J Pharmacol Exp Ther.

[20] Xiong JP, Stehle T, Diefenbach B, Zhang R, Dunker R (2001). Crystal structure of the extracellular segment of integrin αVβ3.. Science.

[21] Suzuki T, Seth A, Rao R (2008). Role of phospholipase Cγ-induced activation of protein kinase Cε (PKCε) and PKCβI in epidermal growth factor-mediated protection of tight junctions from acetaldehyde in Caco-2 cell monolayers.. J Biol Chem.

[22] Shiba T, Nishimura D, Kawazoe Y, Onodera Y, Tsutsumi K (2003). Modulation of mitogenic activity of fibroblast growth factors by inorganic polyphosphate.. J Biol Chem.

[23] Park KJ, Gaynor RB, Kwak YT (2003). Heat shock protein 27 association with the IκB kinase complex regulates tumor necrosis factor α-induced NF-κB activation.. J Biol Chem.

[24] Ni J, Chen SF, Hollander D (1996). Effects of dextran sulphate sodium on intestinal epithelial cells and intestinal lymphocytes.. Gut.

[25] Alex P, Zachos NC, Nguyen T, Gonzales L, Chen TE (2009). Distinct cytokine patterns identified from multiplex profiles of murine DSS and TNBS-induced colitis.. Inflamm Bowel Dis.

[26] Melgar S, Karlsson A, Michaëlsson E (2005). Acute colitis induced by dextran sulfate sodium progresses to chronicity in C57BL/6 but not in BALB/c mice: correlation between symptoms and inflammation.. Am J Physiol Gastrointest Liver Physiol.

[27] Rao NN, Gómez-García MR, Kornberg A (2009). Inorganic polyphosphate: essential for growth and survival.. Annu Rev Biochem.

[28] Brown MR, Kornberg A (2008). The long and short of it - polyphosphate, PPK and bacterial survival.. Trends Biochem Sci.

[29] Tafazoli F, Holmström A, Forsberg A, Magnusson KE (2000). Apically exposed, tight junction-associated β1-integrins allow binding and YopE-mediated perturbation of epithelial barriers by wild-type Yersinia bacteria.. Infect Immun.

[30] Coulson BS, Londrigan SL, Lee DJ (1997). Rotavirus contains integrin ligand sequences and a disintegrin-like domain that are implicated in virus entry into cells.. Proc Natl Acad Sci USA.

[31] Xiong JP, Stehle T, Zhang R, Joachimiak A, Frech M (2002). Crystal structure of the extracellular segment of integrin αVβ3 in complex with an Arg-Gly-Asp ligand.. Science.

[32] Melmed G, Thomas LS, Lee N, Tesfay SY, Lukasek K (2003). Human intestinal epithelial cells are broadly unresponsive to Toll-like receptor 2-dependent bacterial ligands: implications for host-microbial interactions in the gut.. J Immunol.

[33] Otte JM, Cario E, Podolsky DK (2004). Mechanisms of cross hyporesponsiveness to Toll-like receptor bacterial ligands in intestinal epithelial cells.. Gastroenterology.

[34] Vavricka SR, Musch MW, Chang JE, Nakagawa Y, Phanvijhitsiri K (2004). hPepT1 transports muramyl dipeptide, activating NF-κB and stimulating IL-8 secretion in human colonic Caco2/bbe cells.. Gastroenterology.

[35] Nishimura T, Andoh A, Nishida A, Shioya M, Koizumi Y (2008). FR167653, a p38 mitogen-activated protein kinase inhibitor, aggravates experimental colitis in mice.. World J Gastroenterol.

[36] Tao Y, Drabik KA, Waypa TS, Musch MW, Alverdy JC (2006). Soluble factors from *Lactobacillus* GG activate MAPKs and induce cytoprotective heat shock proteins in intestinal epithelial cells.. Am J Physiol Cell Physiol.

[37] Rao NN, Roberts MF, Torriani A (1985). Amount and chain length of polyphosphates in *Escherichia coli* depend on cell growth conditions.. J Bacteriol1.

[38] Rao NN, Kornberg A (1996). Inorganic polyphosphate supports resistance and survival of stationary-phase Escherichia coli.. J Bacteriol.

[39] Kim KS, Rao NN, Fraley CD, Kornberg A (2002). Inorganic polyphosphate is essential for long-term survival and virulence factors in Shigella and Salmonella spp.. Proc Natl Acad Sci U S A.

[40] Rashid MH, Rumbaugh K, Passador L, Davies DG, Hamood AN (2000). Polyphosphate kinase is essential for biofilm development, quorum sensing, and virulence of *Pseudomonas aeruginosa*.. Proc Natl Acad Sci U S A.

[41] Tinsley CR, Gotschlich EC (1995). Cloning and characterization of the meningococcal polyphosphate kinase gene: production of polyphosphate synthesis mutants.. Infect Immun.

[42] Berg DJ, Davidson N, Kühn R, Müller W, Menon S (1996). Enterocolitis and colon cancer in interleukin-10-deficient mice are associated with aberrant cytokine production and CD4(+) TH1-like responses.. J Clin Invest.

